# Lactate and Cancer: Revisiting the Warburg Effect in an Era of Lactate Shuttling

**DOI:** 10.3389/fnut.2014.00027

**Published:** 2015-01-05

**Authors:** Matthew L. Goodwin, L. Bruce Gladden, Maarten W. N. Nijsten, Kevin B. Jones

**Affiliations:** ^1^Department of Orthopaedics, University of Utah, Salt Lake City, UT, USA; ^2^Huntsman Cancer Institute, Salt Lake City, UT, USA; ^3^School of Kinesiology, Auburn University, Auburn, AL, USA; ^4^Department of Critical Care, University Medical Center Groningen, Groningen, Netherlands

**Keywords:** glucose, LDH, metabolism, lactate shuttle, oxygen, VEGF, HIF-1α

## Introduction

Despite causing over half a million deaths per year, cancer continues to elude our understanding and evade our therapeutic approaches. Our comprehension of cancer metabolism lags woefully behind other areas of cancer research. Starting with the seminal experiments done in the 1920s by the Cori’s and Warburg (1, 2), tumors are often described as being glucose avid tissues that produce lactate despite adequate oxygen (O_2_) tension (i.e., the “Warburg Effect”). Only recently, we have begun to understand that tumor cell metabolism is significantly more complicated. Specifically, insight from the study of lactate metabolism has shed light on the peculiar metabolic nature of tumor cells. Here, we present a brief overview of some of the recent developments in the ever expanding literature on lactate metabolism and cancer.

## Lactate Metabolism

First discovered in 1780 by Carl Wilhelm Scheele, lactate was found to be elevated in the muscles of hunted stags in 1808 (3). Later, experiments by Pasteur (4), Meyerhof (5), and A.V. Hill (6) led to widespread understanding of the glycolytic pathway and the notion that lack of O_2_ led to fermentation and lactate accumulation. Out of this early work grew the idea that lactate was a waste that must be cleared from the muscles and blood, preferably by being converted to glucose in the liver via the Cori cycle. However, over the last 50 years, it has been demonstrated in numerous experiments that lactate is both a potent fuel and signaling molecule, and it is constantly being produced and circulated throughout the body, often when there is adequate O_2_ (7). Despite this evidence, lactate as a “hypoxic waste product” is still erroneously taught in many medical schools to this day.

While lack of adequate O_2_ forbids the continuation of oxidative phosphorylation, the notion that lactate production was solely the result of inadequate O_2_ began to change in the 1960s. In a series of elegant experiments in dog muscle, Wendell Stainsby’s group provided evidence that the lactate-releasing canine muscle was not dysoxic (8). Despite criticism over some of his techniques the concept proved correct: lactate formation due to lack of O_2_ is often the exception rather than the rule, even in critically ill patients. The lactate dehydrogenase (LDH) reaction is a rapid, near-equilibrium reaction that lies heavily in the direction of lactate; any time glycolysis is active, lactate is formed and equilibrates with local lactate gradients. Lactate equilibrates mainly by diffusing across membranes via monocarboxylate transporters (MCTs). In lactate-producing tissues or situations, this often means exporting lactate into circulation, where both local and distant tissues can take it up and use it as a fuel.

**Figure 1:**
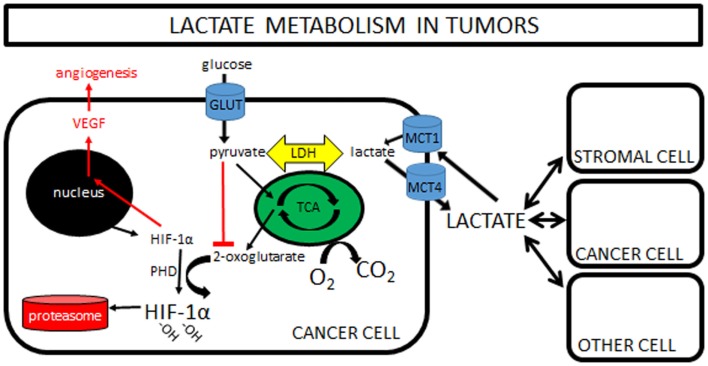
**Lactate metabolism in tumors: a simplified cartoon showing lactate being shuttled to and from cancer cells and its potential role as a signaling molecule in driving angiogenesis**. Increasing pyruvate inhibits formation of 2-oxoglutarate, with the net effect of less degradation of HIF-1α in the proteasome and increased VEGF and angiogenesis. Note that pyruvate can be increased by hypoxia “backing up” the TCA cycle, or by importation of lactate via MCT1s. Some lactate shuttling likely is present between cancer cells and (1) other cancer cells within the tumor, (2) tumor stromal cells, and/or (3) non-tumor cells both local and distant from the tumor. Note that the traditional “Warburg Effect” describes tumors relying heavily on glucose uptake (via GLUTs) with subsequent lactate exportation (via MCT4s) in normoxia. A “Reverse Warburg Effect” describes lactate production from stromal cells, which is then taken up and used by local cancer cells. Some unique combination of these pathways is likely present within each tumor, highlighting the need for further *in vivo* experimentation. (Note that MCTs can transport lactate either direction; MCT1s are typically expressed in cells importing lactate, while MCT4s are expressed in cells exporting lactate.)

This observation that lactate is constantly being produced and consumed formed the basis of the cell-to-cell lactate shuttle, a hypothesis originally introduced by George Brooks in 1984 (9). His widely accepted hypothesis posits that lactate is the key intermediate metabolite in whole body metabolism. It is well described in the literature that lactate can readily replace glucose as a fuel for almost all cells of the body (any cell with mitochondria), including heart, liver, muscle, and even brain (10). So well-supported in so many different experimental settings is his hypothesis that it can now firmly be called “Lactate Shuttle Theory” (11). Finally, more recent work has shown that lactate is also a potent signaling molecule, triggering the stabilization of hypoxia inducible factor-1α (HIF-1α), and subsequently increasing expression of vascular endothelial growth factor (VEGF), resulting in angiogenesis (Figure 1). This new understanding is only now beginning to be explored in tumor models (see Lactate and Cancer: Convergence and Recent Studies below).

## Tumor Metabolism

Unlike lactate metabolism, the study of tumors dates back to as long ago as 1600 BC with descriptions of breast masses (12). However, the modern era of tumor metabolism began in the 1920s with experiments by the Cori’s and Otto Warburg (1, 2). Briefly, the Cori’s showed that the axillary vein draining a hen wing with a sarcoma had a higher lactate and lower glucose when compared to the non-tumor limb. Taking a similar approach, Warburg et al. (2) measured arteriovenous (a–v) differences across tumor beds in rat tumor models. He showed that the vein always had more lactate and less glucose than the artery feeding the tumor, suggesting a net lactate output in presumably normoxic tumor beds. These early investigators clearly recognized the “Warburg Effect,” the “unusual” behavior whereby tumors produce lactate in a normoxic environment.

Do all tumors exhibit the Warburg Effect? In the current era of genomics and the understanding that cancer represents hundreds or thousands of different genotypes, it seems unlikely that all tumor cells would behave in an identical manner. Even in cells of the same clonal expansion, the metabolism of any one cell will vary depending on its local microenvironment. For example, cancer cells at a hypoxic core might use glucose and produce lactate, while cells on the periphery, close to a robust vascular and O_2_ supply, might take up this lactate and oxidize it as a fuel. This concept of shuttling lactate between cancer cells was first introduced by Sonveaux et al. (13). A similar case has been made for a “Reverse Warburg” effect, whereby stromal cells are proposed to produce lactate that tumor cells then take up and oxidize (14).

## Lactate and Cancer: Convergence and Recent Studies

In an insightful commentary on the work done by Sonveaux et al. (13), Semenza poignantly questioned, “Was there any precedent that should have alerted us to this symbiotic relationship between aerobic and hypoxic cancer cells? Of course: the well known recycling of lactate in exercising muscle” (15). Cancer cell metabolism has long been investigated in cultured cells *in vitro*, typically with non-physiological conditions (e.g., media with 25 mM glucose instead of 5 mM for cells that have been passaged many times) that hinder translation to therapeutic models. While great insight can be gained from experiments *in vitro*, it is critical that studies also examine tumors in the context of their local microenvironment. We propose that more studies of animal tumor models *in vivo* are needed to bridge the current gap from bench to bedside.

While one can debate which cells may or may not exhibit a Warburg Effect (16), it is clear that many tumors are glucose avid [the basis of positron emission tomography (PET) scans] and subsequently produce lactate. In a provocative piece in 2009, Nijsten and van Dam (17) presented a hypothetical treatment whereby glucose might be systemically lowered and lactate provided exogenously. If tumors are glucose consumers/lactate producers *and* almost all other tissues in the body can actively take up and use lactate as a fuel, why not systemically induce hypoglycemia to starve tumor cells while providing lactate as a salvage fuel for other tissues? While the idea of inducing hypoglycemia *in vivo* in this manner seems daunting, proof of concept for this idea came from a recent case report out of their hospital (18). This case reports on a patient who walked into the emergency department with extreme hypoglycemia [(glucose) = 13 mg/dL). Not only was he neither comatose nor dead but also was he alert and oriented. Upon first sampling, his blood lactate was 25 mM, likely serving as a salvage fuel for his vital organs, particularly his brain. Work to investigate this concept of a “lactate-protected hypoglycemia” should be pursued.

Finally, lactate also has a role as a potent signaling molecule. This is of particular interest in tumor metabolism, as high-lactate levels are often associated with a worse prognosis [e.g., Ref. (19)]. One proposed mechanism for this poor prognosis is increased angiogenesis. Any change in lactate immediately equilibrates with pyruvate through LDH and vice versa. Accumulating pyruvate inhibits the formation of 2-oxoglutarate, the molecule responsible for targeting HIF-1α for degradation in the proteasome. When lactate (pyruvate) levels increase, HIF-1α drives angiogenesis via VEGF expression. It should be emphasized that HIF-1α stabilization can be driven by lactate or hypoxia independently (Figure 1).

This lactate-to-VEGF pathway has been shown to be independent of O_2_ tension and appears to be an appealing target for potential anti-tumor therapies. De Saedeleer et al. (20) showed that oxidative tumor cells *in vitro* activate HIF-1α via importation of lactate. In another study in glycolytic glioma tumor cells *in vitro*, lactate exposure increased HIF-1α levels independent of hypoxia (21); in a similar study *in vivo* (mice), intraperitoneal lactate administration enhanced xenografted tumor growth, metastasis, and vascularity (22). Finally, another group inhibited MCTs in Lewis Lung carcinoma mice, driving a twofold reduction in vascularity in <2 weeks (13).

In an effort to understand the role of lactate in the tumor microenvironment, the KB Jones Lab at Utah introduced a new transgenic mouse model [alveolar soft parts sarcoma (ASPS)]. In this model, the ASPS oncogene was bred into mice that subsequently developed tumors that were indistinguishable histologically from human ASPS tumors. These vascular tumors had high levels of HIF-1α despite being normoxic throughout. Remarkably, these tumors demonstrated a dramatic increase in vascularity when the mice were given daily intraperitoneal injections of lactate for 2 weeks. Perhaps most interesting was the finding that these tumors formed only within the cranial vault in mice, which was also the area of highest lactate concentration in the mouse (23).

## Conclusion and Future Directions

Current understandings of lactate and tumor metabolism are now converging, seemingly providing as many questions as answers. Lactate is both a potent fuel (oxidative) and signaling molecule (angiogenesis) in most tissues throughout the body. Early work in tumor models suggests that lactate may be either generated and exported or imported and used as a fuel and potent signaling molecule. Lactate-protected hypoglycemia may be a viable strategy in tumors that exhibit a “Warburg Effect,” while MCT inhibitors may be useful in tumors whose angiogenesis is driven by lactate (e.g., ASPS). Most tumors likely lie somewhere between these two extremes, and either or both may soon serve as important adjuvant therapies. It is critical that more studies investigate the metabolic behavior of specific tumors with models *in vivo*. Only when we understand the metabolic behavior of tumors *in vivo* can we then begin to understand how to effectively target them therapeutically.

### Conflict of Interest Statement

The authors declare that the research was conducted in the absence of any commercial or financial relationships that could be construed as a potential conflict of interest.
